# Transition to parenthood and mental health at 30 years: a prospective comparison of mothers and fathers in a large Brazilian birth cohort

**DOI:** 10.1007/s00737-018-0935-x

**Published:** 2018-12-06

**Authors:** R. M. Pearson, Iryna Culpin, C. Loret de Mola, L. Quevedo, J. Murray, A. Matijasevich, K. Tilling, F. C. Barros, A. Stein, B. L. Horta

**Affiliations:** 1 Centre for Academic Mental Health, Population Health Sciences, Bristol Medical School, University of Bristol, Oakfield House, Bristol, BS8 2BN UK; 2 Postgraduate Program in Epidemiology, Federal University of Pelotas, Pelotas, Brazil; 3 Nursing Department, Universidade Federal de Pelotas, Pelotas, Rio Grande do Sul Brazil; 4 Health and Behaviour Postgraduate, Universidade Católica de Pelotas – UCPel, Pelotas, RS Brazil; 5 Departamento de Medicina Preventiva, Faculdade de Medicina FMUSP, Universidade de São Paulo, Sao Paulo, SP Brazil; 6 Department of Psychiatry, Medical Sciences Division, University of Oxford, Oxford, UK; 7 MRC/Wits Rural Public Health and Health Transitions Research Unit (Agincourt), School of Public Health, Faculty of Health Sciences, University of the Witwatersrand, Johannesburg, South Africa

**Keywords:** Brazil, Parental mental health, Parenthood, Epidemiology

## Abstract

Parenthood represents a major biological, social and environmental life change. Mental health disorders are common in parents and impact both the parent and their offspring. However, the relationship between parenthood and mental health and the direction of these effects are poorly understood. Longitudinal data from the Pelotas 1982 birth cohort, Southern Brazil, on 3701 individuals was used to investigate the association between number of children by age 30 years and mental health disorders using DSM-IV diagnoses at age 30 years, suicidal risk and the change in symptoms using repeated measures (using the SRQ-20) from age 19 to 30 years. Mothers, but not fathers, with higher number of children by age 30 years, were at a substantially increased risk of a wide range of mental health disorders compared to women with no children. There was evidence that motherhood was associated with an increase in symptoms over time rather than higher symptoms at baseline. Younger age at first child was also a risk factor for mental health disorders. Mothers, particularly those with multiple children, are at risk of a wide range of mental health disorders. The mechanisms to explain these risks are yet to be elucidated; however, the risk of mental health disorders was not replicated in fathers, which would be expected if residual confounding explained observed associations. Thus, multiparous mothers represent a high-risk group and should be prioritised for supportive interventions.

**Electronic supplementary material:**

The online version of this article (10.1007/s00737-018-0935-x) contains supplementary material, which is available to authorized users.

## Introduction

The transition to parenthood represents a major life change, with substantial impact on financial, social–emotional and physical daily functioning. The incidence of mental health problems rises substantially in child-rearing years, particularly for females (Thapar et al. 2012), who carry the physical burden and, in most cultures, take on the majority of child-rearing. Transition to motherhood is associated with a number of lifestyle changes during a relatively short period of time and includes a variety of biological and psychosocial challenges (e.g. pregnancy, childbirth and taking care of the child, career changes and employment breaks), which may contribute to the female vulnerability to mental health disorders (Goncalves et al. 2016).

The risk of mental health disorders is known to be high amongst parents, and parental mental health disorders are known to be associated with substantial risk of developmental problems in the offspring (Stein et al. 2014). However, several questions remain. Firstly, it is unclear how the transition to parenthood influences diagnostic categories other than depression, which has been the dominant focus of research and intervention to date. Secondly, the direction of the association is not clear, and, for many parents, mental health disorders may precede parenthood (Patton et al. 2015). Longitudinal investigation of the change in mental health, from before the child is born until several years afterwards, is needed to address this question. Finally, comparing the impact of parenthood on mothers and fathers separates the physical burden (which only impacts mothers) from socioemotional and financial changes (which impact both parents to varying degrees) and from shared confounding. Little, if any, longitudinal research exists in fathers or comparing mothers and fathers.

Finally, it is unclear whether the number of children is important. If any risk is associated with the life change of parenthood per se, the number of children may not matter, and the risk will be manifested when women and men make the transition from non-parenthood to parenthood. However, if the demands of parenthood are important, then the risk should increase in a dose–response fashion with increasing number of children being associated with greater load in parental tasks and responsibilities and, thus, greater risk of mental health difficulties. Previous cross-sectional research has linked multiparity with elevated risk of maternal mental health problems; however, evidence is inconsistent (Howard et al. 2014; Ross et al. 2010). It is also unclear whether this risk is due to the characteristics of mothers who have multiple children or increased mental health risk as a consequence of having multiple children. Multiparity is also difficult to separate from young maternal age at giving birth to a first child (which is also linked with mental health risk; Howard et al. 2014).

The 1982 birth cohort, based in south Brazil, provides unique longitudinal data to answer the following questions:

1. Is there an association between parenthood and mental health risk across diagnostic categories at age 30 years? Is this the same in mothers and fathers?
2. Is parenthood at age 30 years associated with higher symptoms compared to parenthood at age 19 years, and is parenthood associated with change in symptoms from age 19–30 years?
3. Does having more children increase the risk in a dose–response pattern, and is this separate from young maternal age?

## Materials and methods

### Data source

In 1982, maternity hospitals in Pelotas, a southern Brazilian city, were visited daily, during the whole year with 99.2% of all births in the city being identified. The 5914 live births whose family lived in urban area of the city were examined, and their mothers were interviewed on sociodemographic and health-related variables by a trained interviewer. These individuals have been followed on several occasions, and further details on study methodology, from previous follow-ups and baseline data, have been published elsewhere (Barros et al. 2008; Victora and Barros 2006). In this study, we primarily utilise data from the follow-up at age 30.2 years (Horta et al. 2015). From February 2012 to February 2013, at a mean age of 30.2 years (hereafter referred to as 30 years), the whole cohort was attempted to be located using multiple strategies. Cohort members were invited to visit our research clinic, where trained personnel interviewed and examined them. The visit included a general interview, physical evaluations and a psychological interview by trained psychologists. We located 4534 cohort members, of which 3701 were interviewed, 467 were living far from Pelotas, 86 refused and another 280, although not having openly refused, did not attend the clinic despite repeated invitations (Horta et al. 2015). We obtained ethical approval for the study from the ethics committee in the ‘Universidade Federal de Pelotas’, all participants gave informed signed consent to take part in the study. During this last follow-up, trained psychologists administered some modules of the Mini-International Psychiatric Interview (MINI) v5.0 validated for Brazil (Amorim 2000) and the Beck Depression Inventory (BDI-II; Beck et al. 1996).

### Materials

#### Exposure variables

The number of children at age 30 years and age at first child was assessed in both male and female cohort participants in the age 30 survey.

#### Outcome variables

##### Mental health

In the last three follow-ups of this cohort, at 18/19, 23 and 30 years, we used the self-reported questionnaire (SRQ-20), validated for Brazil, to assess the presence of common mental health disorders (CMD) (Mari and Williams 1986). Males with a score of ≥ 6 and females with a score of ≥ 8 were considered as positive for CMD with a sensitivity and specificity of 83% and 80%, respectively (Mari and Williams 1986). We calculated the total SRQ-20 score and established the presence of CMD in each follow-up. CMD information, in all three follow-ups, was available for 1447 men and 703 women. We created a variable including only those individuals with a number of CMD episodes.

Using the MINI, only during the last follow-up, we assessed the presence of (i) a major depressive episode during the last 2 weeks, (ii) a lifetime episode of mania/hypomania, (iii) generalised anxiety disorder (GAD) and (iv) social anxiety disorder (SAD). In addition, five questions used in the MINI assessed the presence of suicidal risk.

##### Suicidality

To assess suicidality with and without intent, we asked a number of questions taken from the MINI v5.0 and the BDI-II. A full description of items is provided in Online Resource 1, and discussion with the clinicians who carried out the interviews in Brazilian Portuguese translation confirmed that these items were understood as implying intentionality.

### Confounding variables

Key confounding variables include family income and education levels because both mental health and number of children are socially patterned. In order to avoid introducing bias by adjusting for variables which may be on the causal pathway from exposure to outcome, we adjusted for income and education measured during the early rather than later stages of the study. We also adjusted for relationship status given that it is associated with both mental health and likelihood of having children.

### Statistical analyses

First, we examined the associations between parenthood status and different aspects of mental health at age 30 years using the diagnostic categories on the MINI. We used logistic regression analysis to examine these associations unadjusted and adjusted for baseline family indicators and socioeconomic status. Second, we examined the role of multiparity and the age at first child on the risk of mental health restricting our analyses to those participants who were parents. It is only possible to investigate this association in a sample of parents because non-parents do not have a legitimate value for age at first child. Given that these two variables are relatively interdependent, we used a life-course modelling approach to test specific hypotheses related to the age of mother and the number of children and their impact on mental health. We generated a four-level categorical variable summarising possible combinations of maternal age at first child and number of children:

1. Not young mother/not multiple children = age at first child > 20 years and one child.
2. Young mother/not multiple children = age at first child < 20 years and one child.
3. Not young mother/multiple children = age at first child > 20 years and ≥ two children.
4. Young mother/multiple children = age at first child < 20 years and ≥ two children.

We then used these groups to test the following hypotheses:

#### Hypothesis 1

Only young age is important. In this case, groups 2 and 4 will be the only risk groups because these are the only groups that include young mothers. Group 3 would carry no increased risk from the low-risk group 1 (which we give the notation group 1 = group 3. The risk associated with groups 2 and 4 will be equivalent if the number of children is not important. Therefore, we tested the following constraints: group 1 = group 3 and group 2 = group 4 using a chi-square test to examine whether constrained and unconstrained models fit the data equally well (using the *test* command in Stata). The hypothesis is supported if there is evidence that constrained model does not differ from the unconstrained model.

#### Hypothesis 2

Only having multiple children is important. Therefore, we tested the following constraints: group 1 = group 2 and group 3 = group 4.

#### Hypothesis 3

Both young age and multiple children are important. In this case, group 1 and group 3 will both be the risk groups, and group 4 will represent an accumulative risk. Therefore, we tested the following constraints: group 1 = 0, group 2 = group 3 and group 2 + group 3 = group 4.

These analyses were repeated removing any individuals who had their first child when they were less than 19 years, and results were similar. However, because this resulted in 36% of the sample being dropped, and because the majority of these individuals only had *one* child before age 19 years, we retained the full sample for primary analysis to preserve statistical power and avoid potential bias due to sample restriction.

### Repeated measures

In a third set of analyses, we examined the effect of becoming a parent on the change in SRQ-20 scores from age 19 to 30 years, investigating the influence of parenthood on the intercept (starting point) and slope (change) in SRQ-20 symptoms using linear random effects regression models (*xtmixed* command in Stata).

## Results

At age 30 years, 64% of females (*n* = 1808) and 51% of males (*n* = 1761) had at least one child. Of those males with children, 78% reported that they lived with at least one of their children.

### Motherhood and mental health at age 30 years

There were clear dose–response associations between the number of children by age 30 years and all mental health diagnoses and suicide risk for females (Tables 1 and 2). These associations remained independent following adjustments for confounding variables.

**Table 1:** Logistic regression analyses (odds ratio (OR) and 95% CI) investigating associations between the number of children the women have had from age 19 to 30 years and mental health risk (comparison 1) and comparison of risk in women who get pregnant but do not go onto parenthood due to miscarriage (comparison 2)

Comparison 1	OR (95% CI) for major depression at age 30 years		OR (95% CI) for GAD at age 30 years		OR (95% CI) for SAD at 30 years		OR (95% CI) for manic episodes at 30 years	
	Unadjusted	Adjusted*	Unadjusted	Adjusted*	Unadjusted	Adjusted*	Unadjusted	Adjusted*
Number of children								
0, *n* = 669 (36%)	Ref	Ref	Ref	Ref	Ref	Ref	Ref	Ref
1, *n* = 628 (34%)	1.29 (0.90, 1.86)	0.97 (0.64, 1.47)	1.29 (0.94, 1.78)	1.07 (0.78, 1.46)	0.95 (0.55, 1.63)	0.85 (0.47, 1.52)	2.86 (0.76, 10.82)	2.39 (0.44, 12.73)
2, *n* = 352 (15%)	1.59 (1.06, 2.39)	1.07 (0.66, 1.73)	2.18 (1.55, 3.10)	1.36 (0.95, 1.95)	0.99 (0.52, 1.88)	0.84 (0.42, 1.69)	5.24 (1.38, 19.9)	4.97 (0.95, 26.0)
3, *n* = 132 (7%)	2.84 (1.73, 4.67)	2.16 (1.21, 3.83)	2.39 (1.50, 3.79)	2.31 (1.43, 3.71)	2.30 (1.14, 4.70)	2.05 (0.46, 4.48)	14.76 (3.9, 56.43)	11.44 (2.02, 64.83)
4+, *n* = 76 (4%)	3.87 (2.19, 6.84)	3.70 (1.97, 6.92)	4.18 (2.48, 7.04)	2.53 (1.43, 4.46)	3.70 1.77, 7.77)	2.80 (1.21, 6.50)	12.26 (2.70, 55.90)	15.16 (2.49, 92.14)
Linear *p* value	*p* < 0.001	*p* < 0.001	*p* < 0.001	*p* < 0.001	*p* = 0.001	*p* = 0.016	*p* < 0.001	*p* < 0.001

*Adjusted for family income, maternal education, relationship status and number of miscarriages

*GAD* generalised anxiety disorder, *SAD* social anxiety disorder

**Table 2:** Logistic regression analyses (odds ratio (OR) and 95% CI) investigating associations between the number of children at age 30 years and suicide risk for females (*n* = 1808)

Comparison 1	OR (95% CI) for current suicidal ideation (suicidal thoughts)				OR (95% CI) for ever self-harm/suicidal attempt	
	Without intent		With intent			
	Unadjusted	Adjusted*	Unadjusted	Adjusted*	Unadjusted	Adjusted*
Number of children						
0, *n* = 669 (36%)	Ref	Ref	Ref	Ref	Ref	Ref
1, *n* = 628 (34%)	1.08 (0.64, 1.83)	0.74 (0.41, 1.35)	1.54 (0.84, 2.79)	1.12 (0.58, 2.15)	1.41 (0.91, 2.20)	0.96 (0.58, 1.57)
2, *n* = 352 (15%)	1.70 (0.97, 2.98)	1.08 (0.57, 2.08)	2.38 (1.27, 4.47)	1.75 (0.88, 3.46)	1.61 (0.98, 2.67)	1.21 (0.69, 2.11)
3, *n* = 132 (7%)	3.84 (2.03, 7.27)	2.83 (1.37, 5.82)	4.83 (2.34, 9.97)	3.47 (1.56, 7.74)	2.99 (1.66, 5.40)	2.04 (1.03, 4.05)
4+, *n* = 76 (4%)	5.88 (2.86, 12.06)	4.89 (2.22, 10.73)	8.97 (4.16, 19.30)	5.99 (2.51, 14.30)	4.89 (2.60, 9.23)	4.39 (2.20, 8.76)
Linear *p* value	*p* < 0.001	*p* < 0.001	*p* < 0.001	*p* < 0.001	*p* < 0.001	*p* < 0.001

*Adjusted for family income, maternal education and relationship status

### Motherhood and change in mental health from age 19 to 30 years

There was evidence that motherhood and an increasing number of children (particularly 4+ children) were associated with increased risk of high mental health disorder symptoms (Table 3). In addition, an increasing number of children (as compared to none) were associated more strongly with the change (slope) in symptoms across age 19 to 30 years than the baseline levels (intercept), where increasing number of children was associated with a larger rise in symptoms during this period. These analyses were repeated removing any individuals who had their first child when they were less than 19 years, and results were similar. Given that the observed risk in mental health occurred after the second or third child, the first SRQ-20 was measured before this even in mothers who had their first child before age 19 years.

**Table 3:** Association between the number of children the women have had from age 19 to 30 years and mental health risk on the SRQ-20 (*n* = 716)

	OR (95% CI) for the number of episodes of depression/anxiety on the SRQ							Difference in intercept of SRQ scores at first assessment	Increase in SRQ-20 scores at each assessment from age 19 to 30 years (slope)
	Zero episode	One episode		Two episodes		Three episodes			
		Unadjusted	Adjusted*	Unadjusted	Adjusted*	Unadjusted	Adjusted*	β-Coefficient	β-Coefficient
Number of children									
0, *n* = 669 (36%)	Ref	Ref	Ref	Ref	Ref	Ref	Ref	Ref	Ref
1, *n* = 628 (34%)		1.19 (0.72, 2.0)	1.07 (0.60, 1.90)	0.94 (0.52, 1.67)	0.78 (0.41, 1.50)	3.72 (1.23, 11.21)	2.26 (0.73, 7.00)	0.57 (− 0.27, 1.41), *p* = 0.182	0.01 (− 0.33, 0.34), *p* = 0.958
2, *n* = 352 (15%)		1.62 (0.93, 2.81)	1.69 (0.90, 3.16)	1.16 (0.61, 2.19)	1.03 (0.49, 2.11)	5.73 (1.87, 17.58)	2.88 (0.89, 9.36)	0.23 (− 0.70, 1.17), *p* = 0.621	0.43 (0.05, 0.81), *p* = 0.026
3, *n* = 132 (7%)		2.18 (1.05, 4.52)	1.87 (0.81, 4.35)	3.57 (1.73, 7.34)	2.85 (1.25, 6.47)	10.66 (3.11, 36.42)	4.66 (1.26, 17.27)	1.33 (0.89, 2.57), *p* = 0.036	0.59 (0.08, 1.10), *p* = 0.023
4+, *n* = 76 (4%)		3.96 (1.65, 9.51)	2.52 (0.93, 6.83)	6.10 (2.54, 14.53)	4.00 (1.54, 10.40)	25.36 (7.00, 92.40)	10.20 (2.64, 39.36)	1.22 (− 0.22, 2.67), *p* = 0.098	0.81 (0.21, 1.41), *p* = 0.008
Linear *p* value		*p* = 0.001	*p* = 0.001	*p* < 0.001	*p* = 0.001	*p* < 0.001	*p* = 0.001		

*SRQ-20* self-reported questionnaire validated for Brazil

### Role of number of children and age at first child

There was only weak evidence for an association between age at first child and mental health risk in mutually adjusted model; however, evidence for an association between increased number of children and mental health risk remained (Table 4). In these analyses, it was only relevant to disentangle the role of young maternal age and parity for those who are parents; thus, those without children were excluded. Models testing the independent contribution of young maternal age (< 20) and multiparity (≥ 2 children) provided evidence for a contribution of both variables (i.e. model fit for combined contribution did not differ from that of the unconstrained models).

**Table 4:** Risk factors for mental health outcomes amongst mothers according to age at first child and number of children adjusted for family income, maternal education and relationship status (*n* = 1526)

	Risk of major depression	Risk of GAD	Risk of SAD	Risk of manic episodes	Current suicidal ideation (suicidal thoughts)		Number of SRQ-20 episodes (ordinal logistic regression: OR per 1 more episode)
	OR (95% CI)	OR (95% CI)	OR (95% CI)	OR (95% CI)	Without intent OR (95% CI)	With intent OR (95% CI)	OR (95% CI)
Age at first baby (years)							
25–30	Ref	Ref	Ref	Ref	Ref	Ref	Ref
20–25	1.19 (0.64, 2.2)	1.01 (0.59, 1.74)	0.99 (0.37, 2.64)	1.45 (0.26, 8.09)	1.81 (0.61, 5.32)	2.25 (0.78, 6.49)	0.62 (0.32, 1.20)
< 20	1.37 (0.74, 2.54)	1.62 (0.95, 2.73)	2.04 (0.83, 5.01)	1.23 (0.21, 6.99)	2.95 (1.03, 8.42)	3.62 (1.29, 10.17)	1.25 (0.70, 2.24)
Number of children							
1	Ref	Ref	Ref	Ref	Ref	Ref	Ref
2	1.01 (0.60, 1.67)	1.50 (0.99, 2.28)	0.58 (0.26, 1.30)	1.84 (0.51, 6.67)	1.09 (0.52, 2.24)	1.01 (0.51, 1.99)	1.19 (0.76, 1.89)
3	1.99 (1.01, 3.68)	1.37 (0.77, 2.44)	1.37 (0.58, 3.20)	3.90 (0.95, 5.93)	2.43 (1.10, 5.43)	1.73 (0.77, 3.89)	1.87 (1.05, 3.32)
4+	3.49 (1.75, 6.95)	2.41 (1.26, 4.58)	1.95 (0.78, 4.91)	5.24 (1.11, 24.78)	3.25 (1.29, 8.16)	2.82 (1.15, 6.93)	2.76 (1.45, 5.25)
Test for constraints	Chi2, *p* value	Chi2, *p* value	Chi2, *p* value	Chi2, *p* value	Chi2, *p* value	Chi2, *p* value	Chi2, *p* value
Maternal age but *not* multiparity confers risk	9.71, *p* = 0.008	3.65, *p* = 0.161	1.19, *p* = 0.551	6.29, *p* = 0.043	N/A	9.47, *p* = 0.009	8.07, *p* = 0.018*
Multiparity but *not* maternal age confers risk	10.81, *p* = 0.005	2.86, *p* = 0.240	8.08, *p* = 0.018	0.57, *p* = 0.753	N/A	11.15, *p* = 0.004	11.81, *p* = 0.003*
Maternal age *and* multiparity both contribute risk	0.03, *p* = 0.985	1.02, *p* = 0.603	3.02, *p* = 0.221	3.18, *p* = 0.204	N/A	0.04, *p* = 0.980	4.15, *p* = 0.126*

*Low *p* value means constraints do not fit with observed data patterns

*GAD* generalised anxiety disorder, *SAD* social anxiety disorder, *SRQ-20* self-reported questionnaire validated for Brazil, *N/A* not available

### Comparison with fathers

All analyses were repeated with fathers. There was no evidence for associations between number of children in fathers and any of the mental health outcomes (Table 5). In order to preserve parsimony, we only included the key mental health variables (Table 5), choosing those with greatest statistical power. This decision was driven by the generally lower prevalence of mental health disorders in males, with rarer diagnoses being particularly underpowered.

**Table 5:** Summary of key models in fathers (*n* = 1761) at age 30 years adjusted for income, maternal education and relationship status

	OR (95% CI) for current suicidal ideation (suicidal thoughts)		OR (95% CI) for ever self-harm/suicidal attempt	OR (95% CI) for number of SRQ episodes	OR (95% CI) for depression diagnosis	β-Coefficients for difference in intercept and change in SRQ-20 scores (slope) from age 19 to 30 years	
	Without intent	With intent				Intercept	Slope
Number of children							
0, *n* = 865 (49%)	Ref	Ref	Ref	Ref	Ref	Ref	Ref
1, *n* = 535 (30%)	0.52 (0.24, 1.13)	1.52 (0.71, 3.24)	0.96 (0.52, 1.74)	1.06 (0.82, 1.37)	0.87 (0.45, 1.63)	− 0.08 (− 0.522, 0.36)	0.07 (− 0.12, 0.27)
2, *n* = 258 (15%)	0.79 (0.33, 1.89)	0.66 (0.19, 1.05)	0.87 (0.40, 1.89)	1.08 (0.78, 1.50)	1.59 (0.81, 3.12)	0.09 (− 0.479, 0.65)	0.03 (− 0.22, 0.28)
3, *n* = 72 (4%)	2.08 (0.75, 5.76)	1.70 (0.37, 7.84)	1.66 (0.61, 4.53)	1.63 (0.96, 2.77)	3.42 (1.45, 8.07)	0.15 (− 0.84, 1.13)	0.32 (− 0.12, 0.76)
4+, *n* = 31 (12%)	0.75 (0.10, 5.75)	Too few numbers	1.34 (0.30, 5.96)	1.08 (0.49, 2.39)	1.59 (0.36, 7.10)	− 0.67 (− 2.2, 0.83)	0.57 (− 0.09, 1.23)

*SRQ-20* self-reported questionnaire validated for Brazil

## Discussion

Consistent with existing research, we found evidence that mothers with increasing numbers of children by age 30 years are at a substantially increased risk of a wide range of mental health disorders (Mayberry et al. 2007; Ross et al. 2010).

### Direction of associations

We cannot be sure that this risk was not already present and a potential cause of multiparity (reverse causality). Indeed, mothers who go on to have many children also appear to start off at greater risk. However, our findings suggest that in addition to this baseline risk, mental health symptoms became worse over time following growing numbers of children. This is because the repeated analyses models provide evidence that increasing numbers of children are associated with an acceleration in the rise of mental health symptoms from age 19 to 30 years. It should be noted, however, that adjusting for preexisting mental health problems is problematic in the main analyses because the time that each woman became pregnant varies, and there is always collinearity between previous and current mental health measures. Thus, we adopted a repeated measure approach, using the SRQ data, whereby preexisting mental health is taken into account as the intercept, and this is separated out from slope. The slope (change over time) represents the impact of the number of children after taking into account their baseline (i.e. their preexisting mental health).

### Number of children and age at first child

There was little evidence to suggest that having one or two children was associated with a substantially increased risk of mental health disorders. The risk appears to manifest after three or more children where a clear dose–response pattern was observed. Numbers of children and age at first child are strongly related. When both these variables were included in the same model (sample that included children), there was evidence that the number of children may be the stronger risk factor for most mental health outcomes. However, models generally supported the interpretation that both variables confer risk. Our findings are consistent with existing evidence suggesting that multiparity and age at first child are associated with elevated risks of mental health disorders (Howard et al. 2014).

### Specificity to mothers

The associations appeared to be specific to mothers, with no evidence for any associations observed for fathers. Our finding is in line with previous research suggesting that, unlike motherhood, fatherhood is not associated with increased risk of mental health disorders (Munk-Olsen et al. 2006; Skari et al. 2002). This suggests that the effects are likely to operate through maternal specific pathways, including the physical and psychological demands of motherhood (e.g. high levels of parenting stress, fatigue and physical tiredness; Russell 1974) rather than the more general material and environmental burdens often shared with fathers. Our results may also suggest that mothers are generally more involved in child-rearing activities and that substantial lifestyle changes and psychosocial stressors involved in raising several children pose greater risk for mental disorders in women than in men.

### Strengths and limitations

The strengths of the study include the unique follow-up across the transition to parenthood in both mothers and fathers, as well as rich data on repeated measures of mental health outcomes. In addition, the study examined the effects of multiparity in a context where young maternal age and higher number of children are more common, less socially patterned and more socially acceptable than in the Western world where most previous evidence is derived (Howard et al. 2014).

The findings need to be interpreted in light of several limitations, including restrictions to the generalisability due to the predominantly urban nature of the sample. However, Brazil is a country with wide social disparities and a population-based sample of this size (> 99% of all live births that took place in the city in that year) may be regarded as a representative sample of the city’s population covering the entire social spectrum of the urban population with implications for improved generalisability of our findings (Horta et al. 2015). In addition, we were unable to control for possible unmeasured confounding, such as social support and the nature of the relationship between partners, rather than relationship status (e.g. communication, support, intimacy) that may account for observed associations. Furthermore, the precise timing of each pregnancy, miscarriage or live birth was unknown. Therefore, the role of interbirth intervals or recency of the pregnancy in the mental health outcomes was unclear and merits further investigation. Although we were able to examine the change in symptoms for depression and anxiety to investigate the role of reverse causality, this was not possible for other disorders, particularly suicide risk and manic episodes, where risks were particularly strong. This suggests that for these higher-risk groups, there may be an element of reverse causality, whereby mental health disorders are associated with high-risk behaviours that are associated with higher likelihood of pregnancy, rather than these disorders being a consequence of increasing number of children.

### Mechanisms

It is not possible to claim causality or the direction of effects from these findings alone. However, the overall pattern of findings suggests that decline in mental health (depression and anxiety at least) could be a direct consequence of bringing up multiple children. It is unclear which aspects of motherhood may be responsible for the adverse impact on mental health and should be the subject of future research. There are at least two aspects of multiparty that could negatively impact upon mental health. Firstly, increasing number of children may impose a strain on material and psychological resources within a family, as well as mother’s ability to work, which, in turn, is likely to increase stress and anxiety. Parenting multiple children, including day-to-day feeding, bathing, educating, disciplining and providing emotional support, places increasing demands on parent’s psychological and physical resources. Furthermore, it introduces unique parenting challenges associated with sibling conflict, crowding and practical difficulties associated with engaging in social activities. Increasing number of children may also negatively impact on romantic relationships between parents (Ahlborg and Strandmark 2001).

Secondly, the physical burden of multiple pregnancies and births has hormonal and physiological implications, including likely accumulative increases in women’s BMI (Hardy et al. 2009). This may have a direct impact on mental health through biological mechanisms, as well indirect effect through the impact on body image and self-esteem.

### Implications

As well as having negative consequences for the mother herself, the increased risk of mental health difficulties in multiparous women is also likely to have a negative impact on the children, given the widely documented adverse effects of maternal mental health on a broad range of child outcomes (Stein et al. 2014). The increased risk of mental health problems in multiparous mothers could also explain the increased risk of mental health problems in later born offspring (i.e. maternal mental health may mediate these associations; Lahti et al. 2014). Support services for multiparous women are clearly needed. The number of children is a readily identifiable risk factor. Mothers with three and more children could be offered screening for mental health and necessarily psychological and psychosocial support to alleviate some of the burdens associated with multiparity. This group of mothers may experience unique stressors and, thus, could also be considered a priority for parental support services, such as access to free childcare places and guidance on managing parenting stress. In addition, interventions to promote social support may also be considered, particularly in light of existing evidence suggesting that multiparous women experience less social support (Glazier et al. 2004).

It should be noted that unipolar depression was not the only diagnostic category related to motherhood, which extends to manic episodes, suicide risk and social phobia, all with severe functional consequences. Thus, any existing or future support for mothers needs to consider multiple mental health needs.

## Electronic supplementary material

ESM 1 (DOCX 19 kb)
