# Supplementary material for: Transition to parenthood and mental health at 30 years: a prospective comparison of mothers and fathers in a large Brazilian birth cohort
Source: Arch Womens Ment Health. 2018 Dec 6;22(5):621–9. doi: 10.1007/s00737-018-0935-x (PMC6790207; doi:10.1007/s00737-018-0935-x)
Supplement: Supplementary file 1 — (DOCX 19 kb) [file 737_2018_935_MOESM1_ESM.docx]

**Online Resource 1**

**Transition to parenthood and mental health at 30 years: a prospective comparison of mothers and fathers in a large Brazilian birth cohort**

Culpin, I., PhD;^1^ Loret de Mola, C.,PhD;^2,3^ Quevedo, L. PhD;^4^ Murray, J., PhD;^2^ Matijasevich, A., PhD;^5^ Tilling, K., PhD;^1^ Barros, F.C., PhD;^2,4^ Stein, A., FRCPsych;^6,7^ Horta, B.L, PhD;^2^ Pearson, R.M., PhD.^1^

1. Centre for Academic Mental Health, Population Health Sciences, Bristol Medical School, University of Bristol, United Kingdom.
2. Postgraduate Program in Epidemiology, Universidade Federal de Pelotas, Pelotas, Rio Grande do Sul, Brazil.
3. Nursing Department, Universidade Federal de Pelotas, Pelotas, Rio Grande do Sul, Brazil.
4. Affiliation Health and Behavior Postgraduate Program, Universidade Católica de Pelotas - UCPEL, Pelotas, RS, Brazil.
5. Department of Preventive Medicine, University of São Paulo, Brazil.
6. Department of Psychiatry, Medical Sciences Division, University of Oxford, United Kingdom.
7. MRC/Wits Rural Public Health and Health Transitions Research Unit (Agincourt), School of Public Health.

**Direct correspondence to:** Iryna Culpin, Centre for Academic Mental Health, Population Health Sciences, Bristol Medical School, University of Bristol, Oakfield House, Bristol, BS8 2BN, United Kingdom. E-mail: [iryna.culpin@bristol.ac.uk](mailto:iryna.culpin@bristol.ac.uk); Phone: +44 (0117) 331 0162; Fax: +44 (0117) 331 4026.

**Items to assess suicidality**

To assess suicidality with and without intent we asked a number of questions taken from the MINI v5.0 and the Beck Depression Inventory (BDI). The specific items are listed bellow:

1. During the last month, did you think that you would be better off dead or wish you were dead?
2. During the last month, did you want to harm yourself, to hurt, or to injure yourself?
3. During the last month, did you think about killing yourself?

If YES, during the last month, did you plan on a way of killing yourself?

1. At any time during your life, did you try to kill yourself?

In addition, one item in the BDI-II evaluates ‘thoughts and suicidal wishing’, which was answered by all cohort members. Possible responses to this item were the following:

1. I do not have any thoughts of killing myself.
2. I have thoughts of killing myself, but I would not carry them out.
3. I would like to kill myself.
4. I would kill myself if I had the chance.

It is important to investigate different aspects of suicidal ideation as these may vary across specific clinical disorders and may be associated with differential risks. In addition, it is important to separate current thoughts and intentions from previous attempts. Thus, question 4 (*‘At any time during your life, did you try to kill yourself?’)* was treated as a separate item, and those who responded YES were considered as having a lifetime suicidal attempt. We, therefore, created a ‘current suicidal ideation’ variable comprising three categories: (i) no suicidal ideation; (ii) suicidal thoughts without intent; and (iii) suicidal thoughts with intent.

Those who responded NO to questions 1 to 3 in the MINI v5.0 and reported in the BDI-II that they did not have any thoughts of killing themselves were categorized as ‘no suicidal ideation.’

Suicidal thinking was defined as reporting thoughts of killing oneself on the BDI-II, but not carrying them out; or saying YES *only* to question 1 *(‘During the last month, did you think that you would be better off dead or wish you were dead?’)* and not to the questions below.

Suicidal thinking with intention was defined as saying YES to any question from 2 to 3 in the MINI v5.0; or answering YES to options ‘c’ or ‘d’ on the BDI-II items referring to thoughts and suicidal wishing *(‘I would like to kill myself’ or ‘I would kill myself if I had the chance’).* If any individual met the criteria for suicidal wishing and suicidal thinking/planning at the same time, they were categorized as suicidal thinking/planning group.
